# Risk factors and predictive models for frozen shoulder

**DOI:** 10.1038/s41598-024-66360-y

**Published:** 2024-07-03

**Authors:** Guanjun Sun, Qingshan Li, Yi Yin, Weili Fu, Ke He, Xu Pen

**Affiliations:** 1 Department of Joint Surgery, Suining Central Hospital, Suining City, 629000 Sichuan Province China; 2 Department of Orthopedics, West China Hospital, Sichuan University, Chengdu, 610041 Sichuan Province China

**Keywords:** Diseases, Health care, Medical research, Risk factors

## Abstract

This study aims to explore the risk factors associated with frozen shoulder (FS) and develop a predictive model for diagnosing FS, in order to facilitate early detection of the condition. A total of 103 patients diagnosed with FS and admitted to the Department of Joint Surgery at Suining Central Hospital between October 2021 and October 2023 were consecutively included in the study. Additionally, 309 individuals without shoulder joint diseases, matched for age and gender, who visited the department during the same time, were included as the control group.The complete recording of clinical data for all patients was followed by the utilization of statistical tests such as the Mann–Whitney U test, sample t test, and chi-square test to compare different groups. Additionally, multivariate binary logistic regression analysis was employed to identify risk factors associated with the occurrence of FS in patients, leading to the establishment of a prediction model and derivation of a simplified equation. The diagnostic effectiveness of individual indicators and prediction models was assessed through the use of receiver operating characteristic (ROC) curve analysis. In the sample of 103 individuals, 35 were identified as male and 68 as female, with an average age range of 40–70 years (mean age: 54.20 ± 6.82 years). The analysis conducted between different groups revealed that individuals with a low body mass index (BMI), in conjunction with other factors such as diabetes, cervical spondylosis, atherosclerosis, and hyperlipidemia, were more susceptible to developing FS. Logistic regression analysis further indicated that low BMI, diabetes, cervical spondylosis, and hyperlipidemia were significant risk factors for the occurrence of FS. These variables were subsequently incorporated into a predictive model, resulting in the creation of a simplified equation.The ROC curve demonstrated that the combined indicators in the predictive model exhibited superior diagnostic efficacy compared to single indicators, as evidenced by an area under the curve of 0.787, sensitivity of 62.1%, and specificity of 82.2%. Low BMI, diabetes, cervical spondylosis, and hyperlipidemia are significant risk factors associated with the occurrence of FS. Moreover, the utilization of a prediction model has demonstrated superior capability in forecasting the likelihood of FS compared to relying solely on individual indicators. This finding holds potential in offering valuable insights for the early diagnosis of FS.

## Introduction

Frozen shoulder (FS) is characterized by enduring shoulder pain and restricted mobility^1^. The incidence of FS varies between 2 and 5%, with a higher occurrence in females, particularly among individuals aged 40–70 years^2^. The natural progression of FS is typically self-limiting, with symptoms subsiding within a span of 2 years^3^. Nevertheless, a considerable number of individuals encounter persistent symptoms and functional impairments^4^. In their study, Kim et al.^5,6^ discovered that 41% of the 223 patients examined continued to experience persistent symptoms four years after the initial onset. Additionally, 6% of these patients reported severe pain and loss of function. The symptoms associated with FS have the potential to cause disability in patients and contribute to increased public healthcare expenditures.

Despite being a prevalent condition, there remains ongoing debate surrounding the definition, classification, pathophysiology, diagnosis, natural progression, treatment, and prognosis of FS^4,7^. Typically, the diagnosis of FS relies on the observation of clinical manifestations. The accepted definition of frozen shoulder as stated by American shoulder and elbow surgeons is characterized by pain in the shoulder joint accompanied by restrictions in both active and passive movements, without any abnormalities detected through radiological examination^8^. The consensus reached by the ISAKOS upper limb committee^9^ emphasizes that frozen shoulder is solely used to describe shoulder joint pain and limited range of motion in patients without evident underlying causes. Contributing factors in such patients may include diabetes^10,11^, thyroid disease^12^, Dupuytren contracture^13^, smoking^14^, hyperlipidemia^15^, cardiovascular disease^16^, among others. Secondary shoulder stiffness refers to stiffness of the shoulder joint caused by a specific cause, such as limited range of motion of the shoulder joint due to trauma or surgery.

Despite the existence of numerous studies investigating the risk factors associated with FS, the challenge of early diagnosis persists^17^. Furthermore, a significant proportion of patients exhibit multiple comorbidities^18^. Currently, no research has been conducted to determine whether diverse risk factors can predict the onset of FS. Consequently, the objective of this study is to identify the risk factors associated with FS by means of a case–control study, develop a predictive model, and formulate a simplified equation, with the ultimate aim of offering valuable insights for the early detection of FS.

## Data and methods

### Clinical data

This study employed a case–control design, wherein the case group consisted of 103 consecutive patients diagnosed with FS who were admitted to the Department of Joint Surgery at Suining Central Hospital between October 2021 and October 2023. The case group comprised 35 males and 68 females, aged 40–70 years (mean age: 54.20 ± 6.82 years). All cases underwent shoulder X-ray radiography and MRI examination to assess the condition of the shoulder joint. The control group, on the other hand, consisted of patients without shoulder diseases who visited the same department during the aforementioned period. The control group was matched with the case group in terms of age and gender, with a 3:1 matching ratio, resulting in a total of 309 cases. The similarity in demographics between the two groups was further reinforced by the patients' shared regional origins and similar occupational backgrounds, as indicated in Supplementary Tables 1, 2, and 3.This study received approval from the Ethics Committee of Suining Central Hospital (approval number: LLSLH20220105). Informed consent was obtained from all patients. The data was not publicly available but has been completely anonymised to remove any identifying information in the article.The clinical procedures were carried out in accordance with the Declaration of Helsinki.

### Inclusion criteria

The case group consisted of individuals aged 40–70 years old who met the diagnostic criteria outlined in the literature^9,19^. These criteria included the absence of an obvious cause of shoulder pain, worsening pain after activity, limited range of motion in the glenohumeral joint with external rotation less than 50% of the unaffected side, normal X-ray results, and no other shoulder joint lesions such as rotator cuff tears on MRI. Individuals younger than 40 or older than 70 years old, as well as those who declined to participate, were excluded from the study. The control group was matched with the case group in terms of age and gender, but without shoulder diseases.

### Data collection

The data were gathered and cross-validated by two experienced shoulder surgeons. Prior to inclusion, the X-ray and MRI scans of the shoulder joint were reevaluated to ensure the patient's eligibility. The collection of data encompassed various aspects, including: (1) General information, such as name, age, gender, and BMI; (2) Comprehensive medical history, encompassing conditions like hypertension, diabetes, cardiovascular disease, thyroid disease, Cervical spondylosis, hyperlipidemia, atherosclerosis, and surgical history; (3) Clinical test data, consisting of blood glucose, uric acid, cholesterol (CHOL), triglyceride (TG), high density lipoprotein cholesterol (HDL-C), low density lipoprotein cholesterol (LDL-C),among others.

### Statistical methods

The clinical information collected was subjected to statistical analysis using SPSS25.0 software, with a significance level of α = 0.05 for inter-group comparisons. Descriptive statistics involved the use of the Kolmogorov–Smirnov method to test the normality of quantitative data, and mean ± standard deviation was employed to describe quantitative data that exhibited a normal distribution. For quantitative data that did not conform to a normal distribution, the median (25th percentile, 75th percentile) was used. Categorical data was presented in terms of case number and percentage (%). (1) The independent sample t-test was employed to compare quantitative data between two groups, while the non-parametric rank sum test analysis was utilized for inconsistent conditions. (2) The chi-square test was employed to compare count data between groups. (3) Correlation analysis was conducted using a multivariate binary logistic regression model, with the results expressed as adjusted odds ratios (OR) and corresponding 95% confidence intervals (CI). The independent variable chosen was the indicator that showed significance in the single-factor analysis. (4) Binary logistic regression analysis was employed to derive the β regression coefficient for each independent risk factor, resulting in the formulation of a regression equation and the creation of combined indicators. (5) The receiver operating characteristic curve (ROC) was utilized to calculate the area under the curve (AUC) in order to determine the cutoff value for continuous variables. Furthermore, this approach facilitated the acquisition of diagnostic evaluation results such as sensitivity, specificity, and other relevant metrics.Please note we have moved the section "Ethics approval and consent to participate" to the end of the methods, as per house style.

### Ethics approval and consent to participate

This study received approval from the Ethics Committee of Suining Central Hospital (approval number: LLSLH20220105). Informed consent was obtained from all patients.

## Results

### Characteristics of FS group and inter-group differences

Among the 103 patients diagnosed with FS, the mean age was 54.20 ± 6.82 years, with 35 being male (34.0%). The prevalence of comorbidities in the FS patients was as follows: hyperlipidemia (32.0%), type 2 diabetes (23.3%), cervical spondylosis (18.4%), atherosclerosis (14.6%), hypertension (11.7%), and thyroid disease (2.9%). Additionally, 26 cases (25.2%) presented with two or more comorbidities. Table 1 displays a comparison of indicators between the case group and the control group. A statistically significant difference was observed between the two groups in terms of the incidence of BMI, cervical spondylosis, type 2 diabetes, atherosclerosis, and hyperlipidemia (*P* < 0.05). The case group exhibited a lower BMI compared to the control group, while the four other indicators in the case group were significantly higher. No significant difference was found in the remaining indicators between the two groups (*P* > 0.05).

**Table 1:** Comparison between the case group and the control group.

Indicators	Control group	Case group	t/Z/χ^2^	*P*
Number	309	103		
Age	54.25 ± 6.70	54.20 ± 6.82	0.059^#^	0.953
Gender (male%)	105(34.0)	35(34.0)	0.000*	1.000
BMI (kg/m^2^)	25.03 ± 3.87	23.64 ± 3.33	3.264^#^	**0.001**
Uric acid (umol/L)	307.00(252.00,356.00)	298.00(247.00,351.00)	− 0.872^Δ^	0.383
Blood glucose (mmol/L)	5.56 ± 1.67	5.91 ± 2.33	− 1.411^#^	0.160
CHOL (mmol/L)	5.13 ± 1.10	5.30 ± 0.99	− 1.411^#^	0.159
TG (mmol/L)	1.55(1.03,2.48)	1.54(1.04,2.60)	− 0.632^Δ^	0.527
HDL-C (mmol/L)	1.34(1.14,1.62)	1.35(1.11,1.59)	− 0.343^Δ^	0.732
LDL-C (mmol/L)	2.30 ± 0.79	3.04 ± 0.79	− 0.509^#^	0.611
LDL/HDL	2.24(1.69,2.79)	2.22(1.62,2.96)	− 0.387^Δ^	0.699
Hypertension (%)	35(11.3)	12(11.7)	0.008*	0.929
Cervical spondylosis (%)	1(0.3)	19(18.4)	54.933*	** < 0.001**
Type 2 diabetes (%)	15(4.9)	24(23.3)	30.673*	** < 0.001**
Atherosclerosis (%)	22(7.1)	15(14.6)	5.236*	**0.022**
Hyperlipidemia (%)	54(17.5)	33(32.0)	9.836*	**0.002**
Thyroid disease (%)	1(0.3)	3(2.9)	3.029*	0.082

*: chi-square test; #: t test; Δ: Z tset.

Significant values are in bold.

### Multivariate binary logistic regression analysis results

To investigate the association between BMI, cervical spondylosis, type 2 diabetes, atherosclerosis, and hyperlipidemia as significant indicators of univariate analysis, and their relationship with the occurrence of FS, we employed binary logistic regression. We utilized whether FS occurs as the dependent variable and the five observation indicators as independent variables. The results are presented in Table 2.

**Table 2:** Binary logistic regression analysis of whether FS occurred or not.

Indicators	B	SE	Wald	OR	95%CI		*P*
					Lower limit	Upper limit	
BMI	− 0.452	0.140	10.427	0.636	0.484	0.837	**0.001**
Cervical spondylosis	0.906	0.227	15.987	2.475	1.587	3.859	**0.000**
Type 2 diabetes	0.492	0.113	18.861	1.636	1.310	2.042	**0.000**
Atherosclerosis	0.203	0.111	3.338	1.225	0.985	1.523	0.068
Hyperlipidemia	0.311	0.121	6.592	1.365	1.076	1.732	**0.010**
Constant	− 1.211	0.139	75.896	0.298			0.000

Significant values are in bold.

The logistic regression analysis revealed significant correlations between BMI, cervical spondylosis, type 2 diabetes, and hyperlipidemia with FS (*P* < 0.05). Furthermore, cervical spondylosis, type 2 diabetes, and hyperlipidemia were identified as independent risk factors (OR > 1) that influenced FS, while BMI was identified as an independent protective factor (OR < 1) that influenced FS. Specifically, for every 1 kg/m^2^ increase in BMI, the likelihood of patients experiencing FS decreased by 0.3% (OR = 0.636). Additionally, the likelihood of FS in cervical spondylosis patients increased by 1.475 times (OR = 2.475). The likelihood of experiencing FS in individuals with type 2 diabetes increased by a factor of 0.636 (OR = 1.636). Similarly, the likelihood of FS in patients with hyperlipidemia increased by a factor of 0.365 (OR = 1.365). It is worth noting that atherosclerosis does not emerge as an independent influencing factor for FS, as indicated by a *p*-value greater than 0.05.

### Establishment of prediction model and its diagnostic efficacy

**Figure 1:**
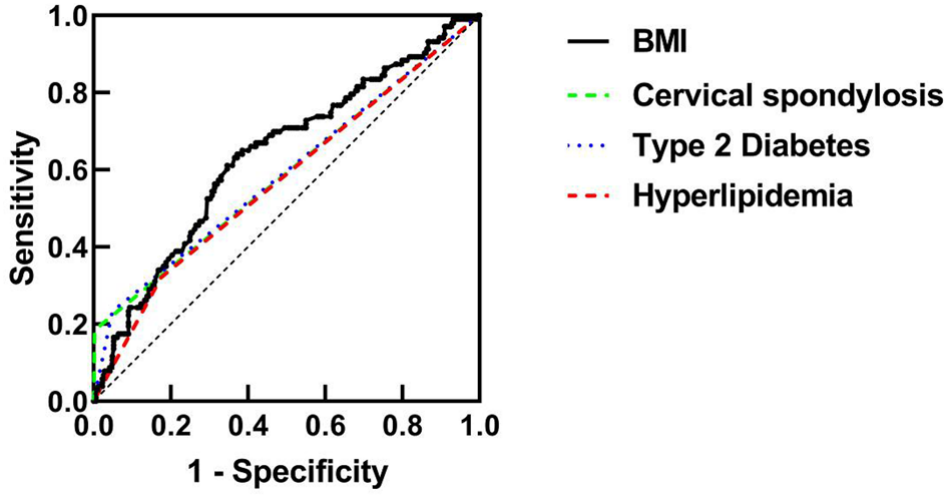
ROC curve analysis.

To enhance the diagnostic significance of BMI, cervical spondylosis, type 2 diabetes, and hyperlipidemia in relation to the incidence of FS, the implementation of ROC curve analysis was employed, as depicted in Table 3 and Fig. 1. The findings presented in Table 3 reveal that the area under the curve for the BMI indicator is 0.637, with a standard error of 0.032, *P* = 0.000, and a 95% confidence interval of (0.574–0.700), suggesting that BMI is not an effective diagnostic tool for FS. The diagnostic performance is optimized by selecting the diagnostic cutoff point as the maximum value of the diagnostic index, which corresponds to a sensitivity of 0.628, a specificity of 0.1, and a diagnostic cutoff point of 23.775 kg/m^2^. Moreover, the diagnostic effectiveness of cervical spondylosis, type 2 diabetes, and hyperlipidemia in diagnosing FS independently is limited.

**Table 3:** ROC analysis.

Indicators	AUC	SE	*P*	95%CI	Cutoff point	Sensitivity	Specificity
BMI	0.637	0.032	0.000	0.574–0.700	23.775	0.628	0.641
Cervical spondylosis	0.591	0.035	0.006	0.523–0.659	–	0.184	0.997
Type 2 diabetes	0.592	0.034	0.005	0.525–0.660	–	0.233	0.951
Hyperlipidemia	0.573	0.034	0.027	0.507–0.639	–	0.320	0.825

In order to obtain the combined diagnostic value of BMI, cervical spondylosis, type 2 diabetes and hyperlipidemia for the occurrence of FS, we first took the occurrence of FS as a dependent variable, and the four indicators of BMI, cervical spondylosis, type 2 diabetes and hyperlipidemia as independent variables. We conducted binary logistic regression analysis, as shown in Table 4, and obtained the combined indicators of the four indicators. Combined indicators = 4.174 * cervical spondylosis + 1.684 * type 2 diabetes + 0.816 * hyperlipidemia -0.122 * BMI. Through approximate rounding, the simplified equation = 6 * cervical spondylosis + 2 * type 2 diabetes + hyperlipidemia -0.2 * BMI.

**Table 4:** Binary logistic regression analysis of combined indicator.

Indicators	B	SE	Wald	OR	95%CI		*P*
					Lower limit	Upper limit	
BMI	-0.122	0.037	10.749	0.885	0.823	0.952	**0.001**
Cervical spondylosis	4.174	1.052	15.750	64.945	8.268	510.135	** < 0.001**
Type 2 diabetes	1.684	0.388	18.839	5.385	2.518	11.516	** < 0.001**
Hyperlipidemia	0.816	0.294	7.708	2.262	1.271	4.026	**0.005**
Constant	1.270	0.891	2.032	3.562			0.154

Significant values are in bold.

**Figure 2:**
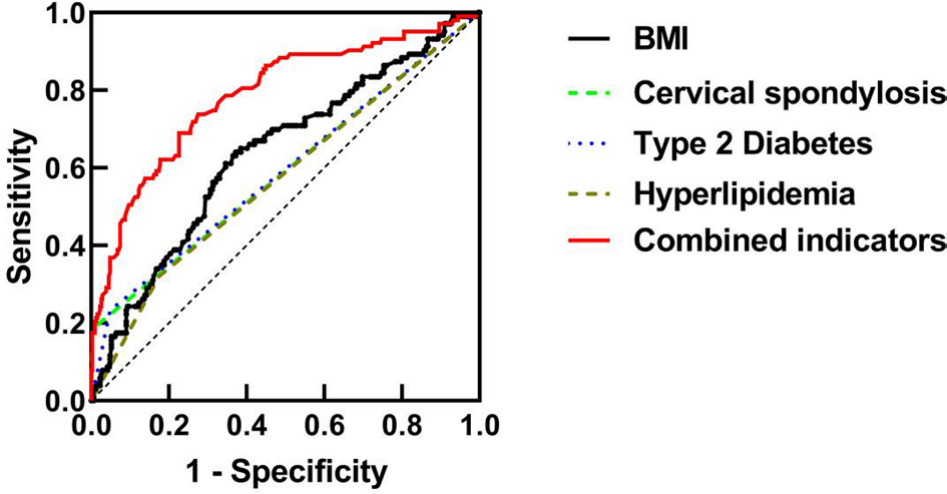
ROC curve analysis of individual indicators and combined indicator.

Subsequently, the ROC curve for the combined indicator was constructed by utilizing the incidence of FS and the combined indicator, as presented in Table 5 and Fig. 2. The outcomes of the combined indicator in Table 5 reveal that the area under the curve for the joint indicator of BMI, cervical spondylosis, type 2 diabetes, and hyperlipidemia is 0.787, with a standard error of 0.028 and a *P*-value of 0.000. The 95% confidence interval is reported as 0.733–0.841, while the sensitivity and specificity are recorded as 0.621 and 0.822, respectively. These findings suggest that the joint indicator demonstrates superior predictive capabilities for the occurrence of FS compared to the single indicator.

**Table 5:** ROC analysis of individual indicators and combined indicator.

Indicators	AUC	SE	*P*	95%CI	Cutoff point	Sensitivity	Specificity
BMI	0.637	0.032	0.000	0.574–0.700	23.775	0.628	0.641
Cervical spondylosis	0.591	0.035	0.006	0.523–0.659	–	0.184	0.997
Type 2 diabetes	0.592	0.034	0.005	0.525–0.660	–	0.233	0.951
Hyperlipidemia	0.573	0.034	0.027	0.507–0.639	–	0.320	0.825
Combined indicator	0.787	0.028	0.000	0.733–0.841		0.621	0.822

## Discussion

Frozen shoulder is a prevalent shoulder joint disorder characterized by a protracted disease course and challenging treatment, resulting in substantial societal and individual burdens^20,21^. During the initial phase of FS, patients typically experience nonspecific pain without significant or mild activity restriction. Hematological tests generally yield normal results, although certain researchers have suggested that early elevation of hypersensitive C-reactive protein may occur^22^. Consequently, the early diagnosis of FS remains a subject of controversy due to the absence of objective criteria^18,23^. Numerous studies have demonstrated the association between FS and various medical conditions such as diabetes, hyperlipidemia, thyroid disease, Dupuytren contracture, cardiovascular disease, and cervical spondylosis^10–16^. However, the practical applicability of these findings in clinical settings remains limited. To address this gap, the present study employed a case–control design, enrolling 103 consecutive patients with FS from the department. Additionally, 309 patients without shoulder joint disease admitted during the same period were matched based on age and gender. The multivariate binary logistic regression analysis revealed that low BMI, cervical spondylosis, type 2 diabetes, and hyperlipidemia were identified as independent risk factors for FS. These factors were subsequently incorporated into a diagnostic prediction model, resulting in the development of simplified equations. The findings indicate that the combined index of BMI, cervical spondylosis, type 2 diabetes, and hyperlipidemia exhibits a favorable predictive capability for the occurrence of FS, as evidenced by an area under the curve of 0.787, a sensitivity of 0.621, and a specificity of 0.822. Consequently, this combined index holds promise as a valuable tool for early clinical diagnosis of FS.

The existing literature has provided limited information on the association between BMI and FS. However, a case–control study conducted by Wei et al.^24^ revealed that an elevated BMI in the case group was not found to be a statistically significant risk factor for FS. Additionally, Pietrzak^16^ has suggested that contemporary society is grappling with a widespread prevalence of obesity, which is strongly linked to the rise in numerous coexisting medical conditions and mortality rates. It is widely acknowledged that metabolic syndrome serves as the primary connection between obesity and the development of diabetes, cardiovascular disease, neurodegeneration, and cancer^25^. Metabolic syndrome arises from endothelial dysfunction caused by hyperinsulinemia, upregulation of pro-inflammatory cytokines, and oxidative stress, resulting in the production of reactive oxygen species, advanced glycation end-products (AGEs), and chronic low-grade inflammatory states^26^. These mechanisms and FS may share common etiological factors. However, a case–control study conducted by Mertens.^27^ arrived at a contradictory finding: low weight and low BMI are identified as risk factors for FS, with the risk of FS increasing by 3% for every kilogram of weight loss. The author expresses uncertainty regarding the underlying cause of this correlation. The findings of this study demonstrate a consistent pattern, wherein a lower BMI serves as an independent risk factor for FS, and each additional unit increase in BMI by 1 kg/m^2^ reduces the likelihood of FS by 0.3% (OR = 0.636). The precise explanation for this association remains unclear, potentially implicating metabolic factors. To gain a more comprehensive understanding, additional investigation is warranted, preferably utilizing multi-center large sample data.

The prevalence rate of FS in individuals with diabetes is significantly higher, ranging from 10–30%, compared to the general population. This condition is considered to be the most influential factor contributing to the development of FS. According to existing literature, the prevalence of FS in diabetes patients is reported to be 1.32–3.69 times higher than in individuals without diabetes^28,29^. A study conducted by Juel et al.^30^ followed 136 cases of type 1 diabetes over a span of 30 years and found that the lifetime prevalence rate of FS in the diabetes group was 76%, while it was only 14% in the control group. Furthermore, there is a positive correlation between the level of cumulative glycosylated hemoglobin and the incidence of FS, indicating that higher levels of glycosylated hemoglobin are associated with an increased risk of developing FS^31^. Certain scholars posit a potential association between AGEs, which are generated via non-enzymatic glycosylation, and the accumulation of these compounds in connective tissues due to their inability to undergo regular degradation and remodeling when they bind to long-lasting proteins. The excessive presence of AGEs can result in pathological collagen cross-linking and structural alterations in tissues, ultimately leading to diminished tissue compliance^32^. This investigation has discovered that type 2 diabetes serves as an autonomous risk factor for FS, with an OR value of 1.636, aligning with previous research findings. Patients with diabetes should be considered as the high-risk group of FS, and may benefit from early diagnosis.

In recent years, there has been a growing body of evidence suggesting that hyperlipidemia is a potential risk factor for FS. In a case–control study conducted by Chang-Meen et al.^33^, involving 1200 patients, it was observed that hypercholesterolemia and inflammatory lipoproteinemia, specifically high low density lipoproteinemia and high non-high density lipoprotein cholesterolemia, exhibited a significant correlation with FS (with an odds ratio ranging from 1.609 to 1.787). Wang et al.^15^ conducted a study utilizing data from the National Health Insurance Research Database to longitudinally track a cohort of 28,748 individuals diagnosed with hyperlipidemia, along with a control group of 114,992 individuals matched through propensity scores, over a period of 5 years. Their findings indicated that the risk of FS in hyperlipidemia patients was 1.5 times greater compared to the healthy control groups. This observation may be attributed to the susceptibility of low density lipoprotein to oxidation and modification by reactive oxygen species, subsequently leading to the activation of endothelial cells within the vascular wall. The activation of endothelial cells results in the upregulation of intercellular adhesion molecule-1 (ICAM-1) and vascular cell adhesion molecule-1 (VCAM-1), thereby attracting inflammatory cells and cytokines associated with inflammation. Additionally, ICAM-1 is implicated in the molecular pathway underlying the occurrence of FS^34^. This investigation establishes hyperlipidemia as a noteworthy risk factor for FS, with an odds ratio (OR) of 1.365, consistent with prior research. Consequently, individuals with hyperlipidemia may derive advantages from early diagnosis.

There is a limited body of research examining the association between cervical spondylosis and FS. Wei et al.^24^ conducted a case–control study and determined that cervical spondylosis is a non-causal risk factor for FS, with a prevalence rate of 23.6% among FS patients compared to 15.3% in the control group. These findings align with previous studies. The authors posit that FS and cervical spondylosis share a similar age of onset, typically occurring after middle age, and suggest a potential causal relationship between the two conditions. The potential for cervical spondylosis to cause FS may be attributed to the presence of lesions in the cervical nerves and blood vessels. Stimulation of the nerves can result in the occurrence of neural radiating pain in the neck and shoulder, subsequently leading to spasms in the surrounding soft tissues of the shoulder joint. The decline in peripheral nerve nutrition, blood circulation, and microcirculation contributes to localized hypoxia in the fiber tissues, an increase in inflammatory substances, worsening of shoulder joint pain, limitation of shoulder joint movement, and the development of shoulder joint stiffness. The study found that the prevalence of cervical spondylosis in the case group was 18.4%, compared to only 0.3% in the control group. Consequently, the logistic regression analysis yielded an odds ratio (OR) value of 2.475, indicating a significant causal risk factor for FS. However, to obtain more accurate evidence, further investigation involving large-scale analysis and in-depth examination of the pathogenesis is necessary.

In conclusion, the case–control study conducted in this research has identified low BMI, cervical spondylosis, type 2 diabetes, and hyperlipidemia as distinct risk factors for FS. Through the development of a diagnostic prediction model and the establishment of combined indicators, it has been demonstrated that the combined indicators of BMI, cervical spondylosis, type 2 diabetes, and hyperlipidemia offer improved predictive capabilities for the occurrence of FS. These findings have potential implications for facilitating early clinical diagnosis of FS. Nevertheless, this study possesses certain constraints. Firstly, it is imperative to acknowledge that this study is confined to a solitary center and encompasses a mere 412 cases. Consequently, the dependability of the outcomes necessitates validation through larger sample sizes obtained from multiple centers. Secondly, while there is a prevailing belief that thyroid disease constitutes a risk factor for FS, the exclusion of thyroid disease patients from the model study due to their limited representation warrants further investigation with more extensive samples.

### Supplementary Information

Supplementary Table 1.

Supplementary Table 2.

Supplementary Table 3.

## Data Availability

The data and materials analyzed during the current study are available from the Supplementary files.
